# Radiolabeled Risperidone microSPECT/CT Imaging for Intranasal Implant Studies Development

**DOI:** 10.3390/pharmaceutics15030843

**Published:** 2023-03-04

**Authors:** Jon Ander Simón, Emilia Utomo, Félix Pareja, María Collantes, Gemma Quincoces, Aarón Otero, Margarita Ecay, Juan Domínguez-Robles, Eneko Larrañeta, Iván Peñuelas

**Affiliations:** 1 Radiopharmacy Unit, Department of Nuclear Medicine, Clinica Universidad de Navarra, University of Navarra, IdiSNA, 31008 Pamplona, Spain; 2 School of Pharmacy, Queen’s University Belfast, Lisburn Road 97, Belfast BT9 7BL, UK; 3 Translational Molecular Imaging Unit (UNIMTRA), Department of Nuclear Medicine, Clinica Universidad de Navarra, 31008 Pamplona, Spain; 4 Department of Pharmacy and Pharmaceutical Technology, University of Seville, 41012 Seville, Spain

**Keywords:** intranasal implant, molecular imaging, SPECT/CT, risperidone, radioiodination

## Abstract

The use of intranasal implantable drug delivery systems has many potential advantages for the treatment of different diseases, as they can provide sustained drug delivery, improving patient compliance. We describe a novel proof-of-concept methodological study using intranasal implants with radiolabeled risperidone (RISP) as a model molecule. This novel approach could provide very valuable data for the design and optimization of intranasal implants for sustained drug delivery. RISP was radiolabeled with ^125^I by solid supported direct halogen electrophilic substitution and added to a poly(lactide-co-glycolide) (PLGA; 75/25 _D,L_-Lactide/glycolide ratio) solution that was casted on top of 3D-printed silicone molds adapted for intranasal administration to laboratory animals. Implants were intranasally administered to rats, and radiolabeled RISP release followed for 4 weeks by in vivo non-invasive quantitative microSPECT/CT imaging. Percentage release data were compared with in vitro ones using radiolabeled implants containing either ^125^I-RISP or [^125^I]INa and also by HPLC measurement of drug release. Implants remained in the nasal cavity for up to a month and were slowly and steadily dissolved. All methods showed a fast release of the lipophilic drug in the first days with a steadier increase to reach a plateau after approximately 5 days. The release of [^125^I]I^−^ took place at a much slower rate. We herein demonstrate the feasibility of this experimental approach to obtain high-resolution, non-invasive quantitative images of the release of the radiolabeled drug, providing valuable information for improved pharmaceutical development of intranasal implants.

## 1. Introduction

Implantable drug delivery systems are capable of providing sustained drug delivery over prolonged periods of time [1,2,3,4,5,6]. The interest in this type of system has been experiencing an increase over recent years due to its potential advantages over conventional drug delivery systems. Implantable systems can be used for either systemic or local drug delivery, with many advantages. First of all, once implanted, these devices area capable of providing unattended sustained drug delivery [7,8,9]. Therefore, patients are not required to rely on continuous and repeated oral intake of medication, which will improve patient compliance [7]. A wide variety of implantable drug delivery systems have been described, including subcutaneous implants [10,11,12,13,14], cardiovascular devices [15,16,17,18,19,20,21], or orthopaedic implants [22,23]. Among these type of implantable devices, intranasal implants have been described for local drug delivery [24]. These devices are normally applied within nasal sinuses after surgery to maintain sinus patency while releasing corticoesteroids to reduce inflammation and reduce polyp recurrence [24].

Intranasal drug delivery has shown potential not only for treating local conditions but to administer drug systemically or even to achieve a more effective delivery of drugs into the brain [25,26]. Most of the formulations developed for intranasal delivery are liquid- or gel-based formulations [27,28,29]. The only type of implantable devices clinically available for intranasal applications are the previously mentioned nasal implants/stents for local drug delivery. Accordingly, there is a clear need for new types of implantable devices that combine the advantages of implantable devices and intranasal drug delivery.

As in vitro release kinetics might differ from in vivo forms, there is a real need for the development of advanced techniques to measure drug release from intranasal implants in vivo. Radionuclide-based non-invasive molecular imaging techniques are currently widely used in the clinical setting for diagnosis of multiple diseases and or pathophysiological altered conditions. In addition, their fully translational nature (“from bench to bedside and back”) has fostered the use either of positron emission tomography (PET) or single-photon emission computed tomography (SPECT) technologies in pharmaceutical development. These techniques can provide three-dimensional, fully quantitative, non-invasive, longitudinal, whole-body images with submillimeter resolution and an extremely high sensitivity that is unsurpassable by any other in vivo imaging technology, given that micro or nanomolar concentrations of radiolabeled molecules in tissues can be detected. In addition, the current state-of-the-art multi-technology devices adapted for small animal imaging combine high-resolution molecular imaging using microPET or microSPECT with anatomical imaging using computed tomography (CT).

The main potential drawback of these techniques is their much lower throughput as compared to others, their cost, and the need for special facilities for the use of radioactive material. However, their fully translational nature and their aforementioned unique characteristics could make them ideal for research, as in the case described in this work.

On the other hand, visible-light in vivo imaging techniques such as bioluminescence and fluorescence are widely available, relatively non-expensive, and can provide a high experimental throughput. However, they have several problems intrinsically bound to the physical nature of the wavelength of visible light photons, including those related with autofluorescence of biological tissues and mainly the lack of real 3D information as visible light photons are significantly absorbed by the tissues, and hence the exact location and intensity of a signal cannot be obtained. Furthermore, these techniques are intrinsically non-quantitative, and only magnitudes such as “relative light units” can be obtained, but there is no way to really obtain fully quantitative values.

This work describes a proof-of-concept study describing the use of intranasal implants for drug delivery using risperidone (RISP) as a model molecule. Applying solid-based radioiodination, we herein radiolabeled RISP with a long-lived radionuclide (^125^I), designed poly(lactide-co-glycolide) (PLGA; 75/25 _D,L_-Lactide/glycolide ratio) (PLGA)-based intranasal microimplants adapted for administration to rats, loaded them with ^125^I radiolabeled RISP, and in vivo imaged microimplants for a month using high-resolution MicroSPECT/CT.

## 2. Materials and Methods

### 2.1. Risperidone Radiolabelling

**Figure 1:**
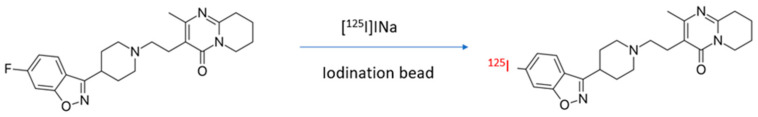
Solid supported ^125^I-radiolabelling of RISP via direct halogen electrophilic substitution.

RISP (Enke Pharma-Tech Co., Ltd., Cangzhou, China) was radiolabeled by direct electrophilic substitution with ^125^I (Figure 1) under oxidative conditions using a modified protocol derived from Saddar et al. [30]. Given the very low solubility of RISP in aqueous solvents, we used Iodination Beads (ThermoFischer Sicentific, Waltham, MA, USA) to achieve mild oxidation conditions and permit a solid-supported reaction in a RISP powder suspension. The radionuclide was chosen on the basis of its long half-life and radioactive properties that could permit follow up of the release of the radiolabeled RISP for more than 1 month. ^125^I has a half-life of 59.49 days, and it decays by electron capture to an excited state of ^125^Te that immediately decays by emission of 35 keV gamma rays.

For the radiolabeling reaction, one Pierce™ iodination bead was pre-wetted in 500 µL of PBS (pH = 6.5) for 5 min, removed from the solution, and dried over filter paper. Thirty milligrams of RISP was weighted in a bottom-flat glass vial, and the iodination bead was added along with 300 µL of fresh PBS and 18,5 MBq of [^125^I]INa solution (Perkin Elmer Inc, Amsterdam, the Netherlands) in <5 µL. The suspension was thoroughly mixed by shaking and left with gentle magnetic stirring for ≈72 h.

### 2.2. Quality Control

The radiolabeling reaction was periodically monitored by radio thin-layer chromatography (radioTLC). For this purpose, samples were taken from the reaction vial just after increasing the stirring speed. Such samples (that contained both the non-dissolved RISP and the reaction solution) were added into 10 µL of an acidified PBS solution (3 mL of HCl 1 M:2 mL of PBS) to dissolve RISP, then seeded at 1 cm from the bottom of a 10 cm iTLC SG strip (Agilent Technologies, Santa Clara, CA, USA) that was developed to a final distance of 8 cm from the origin with 0.9% NaCl. Chromatograms were analyzed using a radioTLC scanner (Scam RAM, LabLogic, Sheffield, UK). The separation method had previously been validated by our group using non-radioactive RISP and iodide samples visualized with a 7.5 g/L KMnO_4_, 50 g/L K_2_CO_3_, and 0.625 g/L NaOH solution as described in the Section 3.

### 2.3. ^125^I-Risperidone Extraction and Purification

At the endpoint of the reaction, the iodination bead was removed from the radiolabeling vial, the suspension was thoroughly mixed and transferred into an Eppendorf tube, the vial was washed twice with 200 µL of PBS, and the three samples were mixed. The overall reaction suspension was centrifuged at 9000 rpm for 1 min, and the supernatant was removed. The solid precipitate was then washed twice with 200 µL of PBS, and the supernatant was analyzed by radioTLC as described above to check for the absence of free [^125^I]I^−^. The precipitate containing ^125^I-labelled RISP (^125^I-RISP) was then dried overnight with gentle shaking at 37 °C in a ThermoMixer C (Eppendorf Thermoshaker, Hamburg, Germany).

### 2.4. Preparation of Microimplants Containing ^125^I-RISP or [^125^I]INa

**Figure 2:**
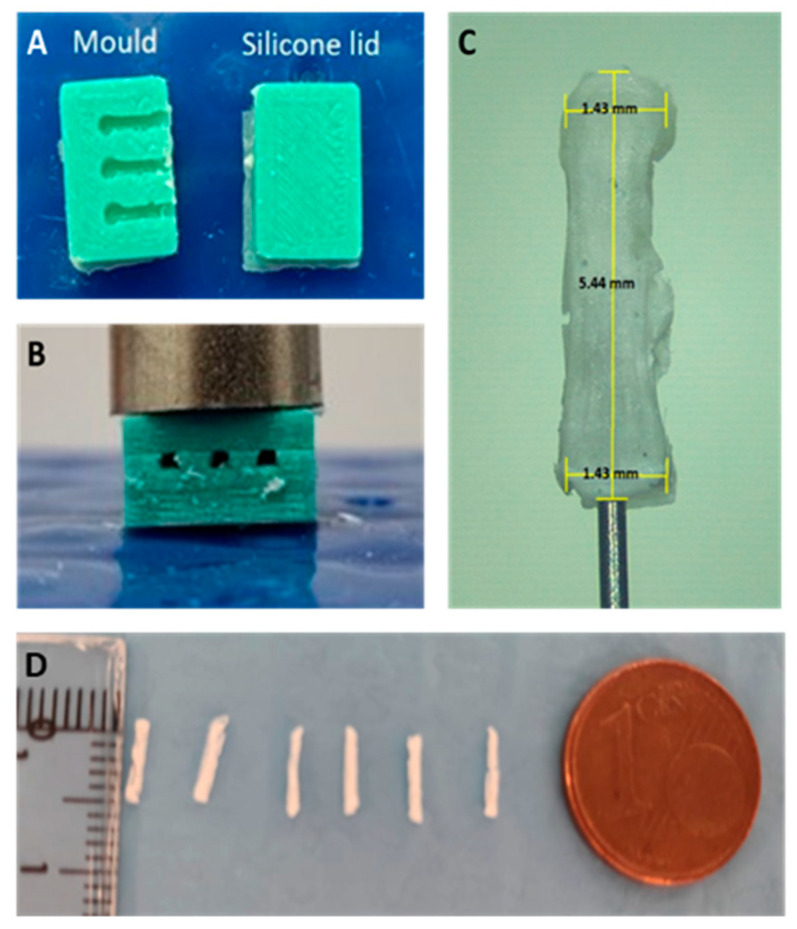
Images showing silicone molds used to prepare intranasal implants (**A**,**B**). Microscopy image of a PLGA implant prepared using the silicone molds (**C**). Size comparison of microimplants (**D**).

To prepare PLGA-based microimplants, silicone molds were used (see Figure 2A,B). These implants were prepared by using a 3D-printed poly(lactic acid) and casting silicone on top as described previously (Figure 2C,D) [11]. The silicone (Xiameter^®^ RTV-4250-S) (Notcutt, Surrey, UK) was prepared by mixing a silicone elastomer with a curing agent (ratio 10:1). The molds contained two parts: a part containing 3 cavities to prepare 3 implants and a lid. Viatel^TM^ DLG 7509 E PLGA (75/25 _D,L_-Lactide/glycolide ratio; M_n_ = 61.9 kDa; M_w_ = 104.2 kDa; Tg = 50 °C; ester end group) (Ashland Specialities Ireland, Mullingar, Ireland) was used to prepare implants. In order to achieve this, approximately 20 mg of the dry solid ^125^I-RISP mixture was dissolved in 70 µL of dichloromethane. Subsequently, 16 mg of PLGA was added to the ^125^I-RISP solution, and the solution was carefully mixed (to avoid bubble formation) for 20 min. Using the silicone mold and a positive displacement pipette, 15 µL of the ^125^I-RISP/PLGA mixture containing around 750 kBq was added to the silicone molds to prepare each microimplant. After 24 h at room temperature, microimplants were taken out from the molds, burrs were carefully removed and longitudinally cut in half, and their activity was measured in a dose calibrator calibrated for ^125^I. Such halves contained 150–225 kBq ^125^I and were rigid enough for intranasal in vivo administration. As controls for in vitro and in vivo release studies, microimplants containing [^125^I]INa were prepared in a similar way but using [^125^I]INa instead of ^125^I-RISP.

### 2.5. In Vitro Release Studies

Microimplants containing either ^125^I-RISP or [^125^I]INa were placed in 2 mL PBS at 37 °C with constant agitation (350 rpm) in a thermomixer. Five microliter triplicate samples were taken at defined time points for up to 30 days and radioactivity measured in a gamma counter (Hidex Automatic Gamma Counter, Turku, Finland) calibrated for^125^I, and percentage release ratios were calculated.

In parallel, the release of unlabeled RISP from PLGA implants in PBS (pH = 6.5) at 37 °C was evaluated using HPLC. For this purpose, an Agilent 1220 Infinity II LC gradient system (Agilent Technologies UK Ltd., Stockport, UK) equipped with a Phenomenex^®^ SphereCloneTM C18 ODS column (150 mm length × 4.60 mm internal diameter, 5 µm particle size) was used. The mobile phase contained a mixture of organic (85% *v*/*v*) and aqueous phases (15% *v*/*v*). The aqueous phase contained 10 mM sodium dihydrogen phosphate buffer. On the other hand, the organic phase contained a mixture of methanol and acetonitrile (75:25% *v*/*v*). RISP detection was carried out at 235 nm.

### 2.6. Animal Studies

Female Wistar rats (212 ± 22.5 g, Harlan Laboratories S.A., Barcelona, Spain) were used. Animals were socially housed on 12 h light–dark cycles under standard conditions in compliance with the current regulation and given free access to food and water.

### 2.7. Microimplant Intranasal Administration and In Vivo Release Studies

**Figure 3:**
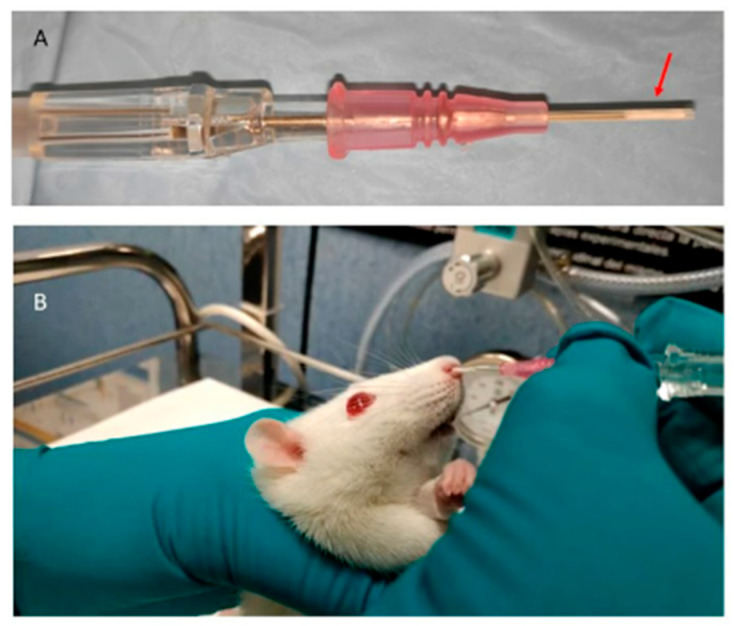
Microimplant (red arrow) placed inside the modified catheter (**A**). Intranasal administration of the implant to the animal (**B**).

Radiolabeled microimplants halves (≈5.5 mm × 0.5 mm) were introduced into a 20 GA 1.1 mm × 30 mm i.v. catheter (Insyte Autoguard BC, BD medical, Madrid, Spain) previously cut at around 15 mm from the hub to flatten the bevel and shorten its length. A total of 7–10 mm of the catheter were slowly introduced through the nostril of anesthetized rats (2% isoflurane in 100% O_2_ gas), the shaft was carefully pushed until the implant was left into the nasal cavity, and then the catheter was removed (Figure 3). Six animals were treated with ^125^I-RISP microimplants, while three were treated with microimplants loaded with [^125^I]INa as controls. The correct placement of the microimplants inside the nasal cavity was verified by microSPECT/CT imaging.

After microimplant administration, in vivo images were acquired just post-administration (day 0) and at 1, 3, 7, 11, 14, 18, 21, and 28 days. SPECT scans were acquired in a U-SPECT6/E-class (MILabs, the Netherlands) using an ultrahigh resolution UHR-RM-1 mm multi-pinhole collimator. Rats were placed prone on the scanner bed under continuous anesthesia with isoflurane (2% in 100% O_2_ gas) to acquire dynamic scans of the head in list mode format over 30 min. Following the SPECT acquisition, and without moving the animals, CT scans were performed to obtain anatomical information using a tube setting of 55 kV and 0.33 mA. All the SPECT images were reconstructed using the ^125^I photopeak centered at 29 keV with a 20% energy window width and using a calibration factor to obtain the activity information (MBq/mL). Finally, attenuation correction was applied using the CT attenuation map. To obtain fully quantitative values (MBq), the system was calibrated using a point source prepared from [^125^I]INa.

Studies were exported and analyzed using the PMOD software (PMOD Technologies Ltd., Adliswil, Switzerland), where fully three-dimensional fused SPECT/CT images were processed. The retention of radioactivity in the microimplant was calculated for each image as follows: a spherical volume of interest (VOI) containing the entire microimplant was drawn over SPECT images using the CT co-registered images as anatomical reference. Then, a semiautomatic delineation tool was used applying a predefined threshold of 1% of the maximum voxel value to obtain a new VOI that delimited the entire signal. Finally, the average value inside the VOI (MBq/mL) multiplied by the volume (mL) was calculated to estimate the amount of radioactivity retained in the microimplant. From these data, the corresponding ^125^I decay correction based on the half-life (T_1/2_) of ^125^I and the time elapsed between the administration and the imaging was applied (A = A_0_ ∗ e(−ln(2) ∗ t/T_1/2_). The percentage of the release was calculated as the inverse of the percentage of the retention detected in each image and considering the retention value in the 1 h post-administration image as the administered dose for each animal.

## 3. Results and Discussion

### 3.1. Reaction Optimization and Radiolabelling Yield

The radiolabeling yield was studied at different time intervals by radioTLC. Before taking a sample for radioTLC, the stirring speed was increased to be able to pipette a representative sample containing both a fraction of the solid (RISP) and the liquid in a suspension. Furthermore, after around 24 h, a solid precipitate could not be seen any more, and the suspension had a milky-turbid visual appearance. At 24 h, the labelling yield was around 50%, and it only increased marginally up to 72 h.

RISP iodination reaction was first tested in an Eppendorf tube. Although the tube was inverted several times every 2 h, a rather large part of the RISP was not in contact with the solution because it precipitated under reaction conditions and the contact of the solid with the iodination bead was not sufficient to get appropriate yields. Radiolabeling was then tested in a flat-bottom 5 mL glass vial that permitted continuous magnetic stirring at low speed for the overall reaction time (up to 72 h). Using the simple approach of combining a surface-based reaction on the iodination beads with a water-insoluble molecule such as RISP, we were able to carry out the radioiodination exchange reaction in suspension. Although the reaction yield was relatively low (around 25%), after extraction, centrifugation, and two consecutive washing steps, virtually all [^125^I]I^−^ was removed from the reaction mixture as determined by the radioTLC measurement of radioactivity in the different supernatants. The extraction and purification processes were optimized to maximize the purity of the final product, not the reaction yield.

RISP has previously been radiolabeled with ^125^I [30], although by direct electrophilic substitution by oxidation with chloramine-T in an alcoholic solution of RISP. Strong oxidation with aggressive reagents such as chloramine-T might produce alterations in the molecule, and this requires a careful optimization of reaction conditions and times. In our hands, mild oxidation with iodination beads produced a smoother and more controllable reaction, albeit the final reaction yield was usually lower. Nonetheless, we decided to use a solid-supported electrophilic substitution and a final extraction step to maximize purity of ^125^I-RISP and not reaction yield. In this way, using high specific activity [^125^I]INa (≈629 GBq/mg), we were able to obtain >99% pure ^125^I-RISP in sufficient amount to make PLGA microimplants containing radiolabeled RISP for in vivo imaging. Given the chemical structure of RISP, we might have considered radiolabeling it with fluorine-18 (a PET radionuclide with a half-life of 109.8 min) producing an identical molecule to the parent one. This is not only a very complex synthesis that has only been described in one paper thus far [31] (and never been used for in vivo imaging), but is also performed with an extremely short half-life radionuclide that could in no way be used for long-term release studies such as the ones presented here.

### 3.2. Quality Control Validation

**Figure 4:**
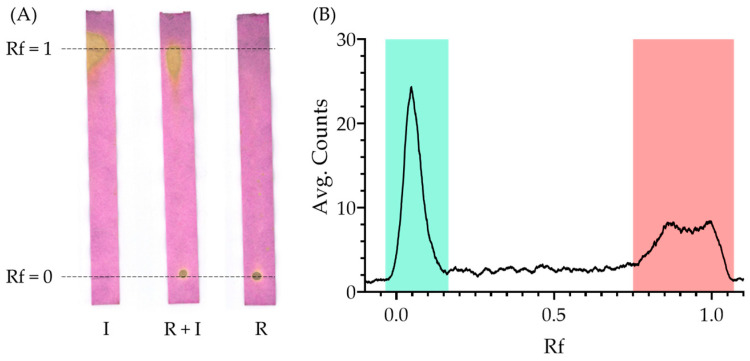
(**A**) Permanganate-stained TLC strips. RISP (R) remained at Rf = 0, while sodium iodide (I) advanced to the front (Rf = 1). The mixture of both compounds (R + I) showed that the mixture did not alter the individual results. (**B**) Representative radioTLC radiochromatogram of radiolabeled ^125^I-RISP at 72 h, before purification.

As can be seen in Figure 4A, using iTLC SG strips developed in saline and stained with permanganate, the mixture of RISP and KI showed no interferences in TLC, and both species could be clearly resolved.

When using radioactive samples for QC of RISP radiolabeling, both species were able to be properly resolved and identified (see a representative radioTLC chromatogram in Figure 4A,B).

### 3.3. In Vivo and In Vitro Release Studies

**Figure 5:**
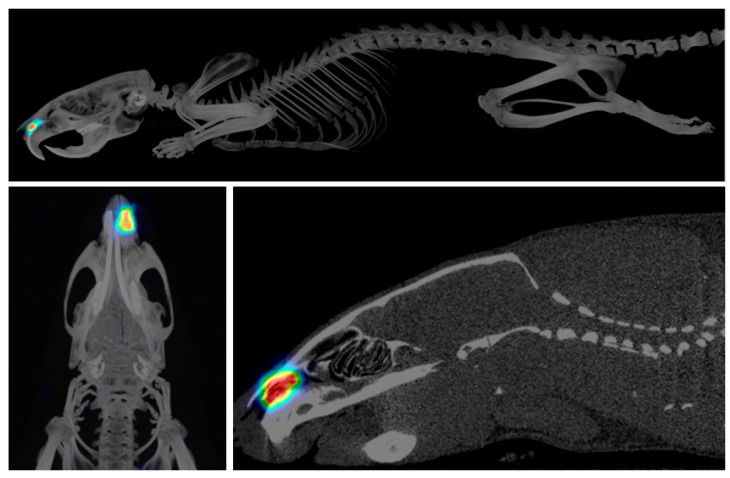
Fused MicroSPECT-CT images showing the location of the microimplant inside the nasal cavity. Hotter colours indicates higher concentration of radioactivity.

**Figure 6:**
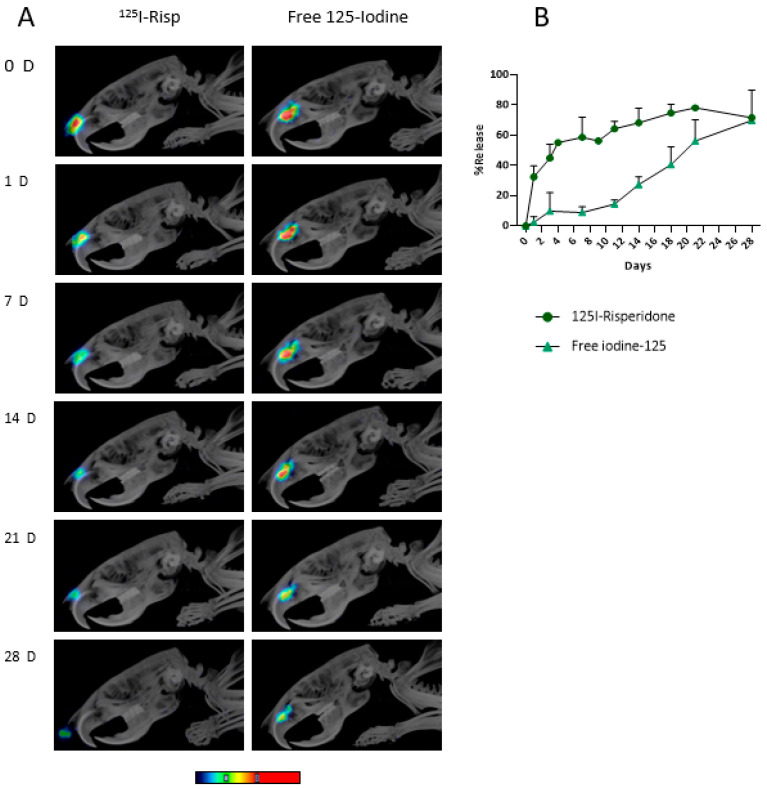
(**A**) Comparative release of radioactivity in vivo from ^125^I-implants. MicroSPECT-CT images clearly show a progressive decrease in the amount of radioactivity in the nasal cavity over time, both for ^125^I-RISP and [^125^I]INa implants. Hotter colours indicates higher concentration of radioactivity. The plot in (**B**) shows comparative quantitative values of radioactivity release as measured in the images on the left (mean ± SD).

SPECT-CT images show the correct placement of the microimplant inside the nasal cavity of the animals (Figure 5), thus demonstrating the feasibility and accuracy of the administration procedure we developed. The sensitivity and resolution of MicroSPECT depends on the radionuclide used and the scanner, but it can be as low as 0.5 mm. Further details of the MicroSPECT/CT system were previously reported by Prieto et al. [32]. Longitudinal images acquired in all animals for around 4 weeks showed that the implants remained inside the nasal cavity for a long time (Figure 6). The amount of radioactivity in the microimplant progressively decreased over time (all data were corrected for the decay of ^125^I and were hence comparable). When comparing the release of ^125^I-RISP and [^125^I]I^−^, a clearly different pattern was seen with faster clearance of the former (Figure 6), probably due to its lipophilic nature. ^125^I-RISP release reached around 45% by day 3 and then steadily but slowly increased up to 80% by day 21. [^125^I]I^−^ release was much slower, and it only accounted for 17% by day 12, and then it increased with a larger slope up to around 45% by day 21. Between 3 and 4 weeks after implantation, the implant moved from its original placement position towards the nostril, probably due to the progressive dissolving of the PLGA matrix (see Figure 6A at 28 days). The in vivo imaging technique used allowed us to even be able see this phenomenon.

**Figure 7:**
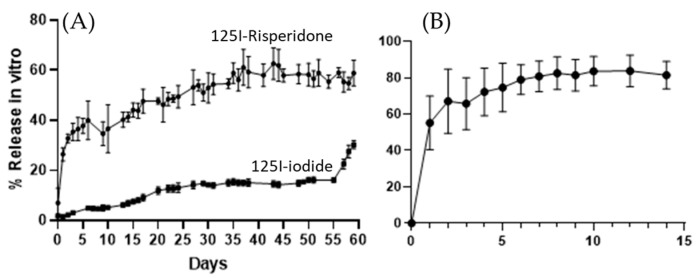
Radioactivity release in vitro from ^125^I-implants are shown in (**A**), while (**B**) shows RISP release from implants as determined by HPLC (mean ± SD).

In vitro release studies of ^125^I-RISP and [^125^I]I^−^ from the microimplants also showed a different pattern, although the different experimental conditions between in vitro and in vivo experiments figures were somewhat different (Figure 7). HPLC release studies of unlabeled RISP implants showed a fast release of RISP in the first 2 days (up to 65%) and then a very slow increase up to 80% by day 7.

All three methods (in vivo SPECT-CT imaging, in vitro radioactivity release studies, and HPLC release studies) showed a fast release of the drug in the first days and then a much steadier increase to reach a plateau-like situation. Release profiles are mostly comparable, but figures and slopes are different for each method, yet all three methods show that RISP can be released from our implants for a long time.

The highly lipophilic drug used in these experiments was slowly but steadily released from the microimplants, while the release of a hydrophilic compound ([^125^I]INa) took place at an slower rate. If both the lipophilic molecule and the ionic one would be equally trapped in the PLGA implant matrix, and their release would only depend on the progressive dissolution of the implants over time in vivo, quantitative values and release profiles of ^125^I-RISP and [^125^I]I^−^ would be similar. Our data show that the lipophilic or hydrophilic nature of the molecule of interest definitely conditions its release (as expected). Consequently, the dissolution of the implants would be responsible to some extent for the release of the loaded test molecule, but the specific physicochemical characteristics of the molecule under study and its interactions with the implant matrix are also of paramount importance, not only in in vitro experiments, but also in vivo, as shown by our images. The exact relationship between the release produced by dissolution of the implant and progressive release from the matrix are difficult to estimate for small ionic molecules (such as iodide) when a matrix as PLGA is used.

It is important to note that ^125^I-RISP was not seen in the brain or any other part of the animal. The reason behind this could be the small amount of radioactivity used in each implant (just a few kBq, as compared to magnitudes in the order of MBq usually used for in vivo imaging in small animals). Furthermore, the passage of iodinated RISP through the nose to brain cribiform plate is unknown, and it might be different to some extent different to that of RISP, leading to a lower entrance into the brain. However, it is important to note that previous studies showed that intranasal RISP formulations showed enhanced drug uptake [33,34,35]. This work describes a new method for RISP radiolabeling and loading into intranasal microimplants. These implants have been designed to be implanted inside the nasal cavity to provide enhanced brain delivery of drugs for potential treatment of schizophrenia. Intranasal implants can revolutionize the treatment of chronic conditions affecting the central nervous system as they can provide sustained drug delivery and enhanced brain targeting. As mentioned previously, nasal stents and nasal drug eluting implants are currently been used to treat nasal polyps [24,36,37,38,39]. Accordingly, they are designed for localized drug delivery. Modifying this type of implant to provide sustained drug release into the brain can be used in the treatment of a wide range of chronic conditions. These devices are capable of providing prolonged drug delivery and, therefore, have potential to improve patient adherence to treatment [40,41,42,43]. This is especially important for schizophrenia treatment, as it has been reported that up to 75% of patients discontinue the treatment within the first year and a half [44]. Non-adherence to treatment has an enormous impact for this patient as it increases the risk of relapse, hospitalization, and even suicide rates [45,46,47,48,49]. Moreover, there is an obvious economic impact for healthcare systems. In the UK, it has been estimated that the cost of relapse per patient can be up to GBP 15,000 per year [50]. This figure is four times higher than the equivalent cost for non-relapse patients [51]. In addition, in order to improve patient compliance, nasal drug delivery will improve brain uptake, minimizing the risk of systemic exposure and potential side effects. Therefore, intranasal implants for the treatment of chronic conditions affecting the central nervous system offer multiple benefits to conventional treatment. However, before these systems can be used, in vivo testing is required. In the present work, we have shown potential alternatives to evaluate in vivo drug release using radiolabeled microimplants containing RISP.

## 4. Conclusions

To the best of our knowledge, this is the first study to describe the use of radiolabeled RISP for the development of intranasal implantable devices. This procedure can be used to evaluate drug release in vitro and in vivo in a simple way. This is especially important for in vivo drug delivery. We herein demonstrate the feasibility of this approach and its application to obtain high-resolution images of the release of the radiolabeled drug from the microimplants for more than one month. Furthermore, the possibility to accurately quantify in vivo the amount of the drug in the target, instead of simply measuring the concentration of the drug or its metabolites in the body fluids (i.e., blood, urine, etc.) as a result of the release, provides very valuable data for the development and fine-tuning of implantable long-term drug release devices. Future studies addressing the relationship between dosage of the number of therapeutic molecules in the brain and blood of the animals and the values quantitatively evaluated by imaging would further help clarify this point.

## Data Availability

Data described in the manuscript, including all relevant images, are available from the corresponding author on reasonable request.
